# Three dimensional microelectrodes enable high signal and spatial resolution for neural seizure recordings in brain slices and freely behaving animals

**DOI:** 10.1038/s41598-021-01528-4

**Published:** 2021-11-09

**Authors:** P. Wijdenes, K. Haider, C. Gavrilovici, B. Gunning, M. D. Wolff, T. Lijnse, R. Armstrong, G. C. Teskey, J. M. Rho, C. Dalton, Naweed I. Syed

**Affiliations:** 1 Faculty of Medicine, Hotchkiss Brain Institute, University of Calgary, 2500 University Dr. NW, Calgary, AB T2N 1N4 Canada; 2 Biomedical Engineering Graduate Program, University of Calgary, 2500 University Dr. NW, Calgary, AB T2N 1N4 Canada; 3 Alberta Children’s Hospital Research Institute, University of Calgary, 2500 University Dr. NW, Calgary, AB T2N 1N4 Canada; 4 Department of Cell Biology and Anatomy, University of Calgary, 2500 University Dr. NW, Calgary, AB T2N 1N4 Canada; 5 Department of Electrical and Computer Engineering, University of Calgary, 2500 University Dr. NW, Calgary, AB T2N 1N4 Canada; 6 Departments of Neurosciences and Pediatrics, University of California San Diego, Rady Children’s Hospital, San Diego, CA USA; 7 Cumming School of Medicine, University of Calgary, 3330-Hospital Drive, NW, Calgary, AB T2N 4N1 Canada

**Keywords:** Neural circuits, Neuronal physiology, Nanobiotechnology, Biomedical engineering

## Abstract

Neural recordings made to date through various approaches—both in-vitro or in-vivo—lack high spatial resolution and a high signal-to-noise ratio (SNR) required for detailed understanding of brain function, synaptic plasticity, and dysfunction. These shortcomings in turn deter the ability to further design diagnostic, therapeutic strategies and the fabrication of neuro-modulatory devices with various feedback loop systems. We report here on the simulation and fabrication of fully configurable neural micro-electrodes that can be used for both in vitro and in vivo applications, with three-dimensional semi-insulated structures patterned onto custom, fine-pitch, high density arrays. These microelectrodes were interfaced with isolated brain slices as well as implanted in brains of freely behaving rats to demonstrate their ability to maintain a high SNR. Moreover, the electrodes enabled the detection of epileptiform events and high frequency oscillations in an epilepsy model thus offering a diagnostic potential for neurological disorders such as epilepsy. These microelectrodes provide unique opportunities to study brain activity under normal and various pathological conditions, both in-vivo and in in-vitro, thus furthering the ability to develop drug screening and neuromodulation systems that could accurately record and map the activity of large neural networks over an extended time period.

## Introduction

**Figure 1:**
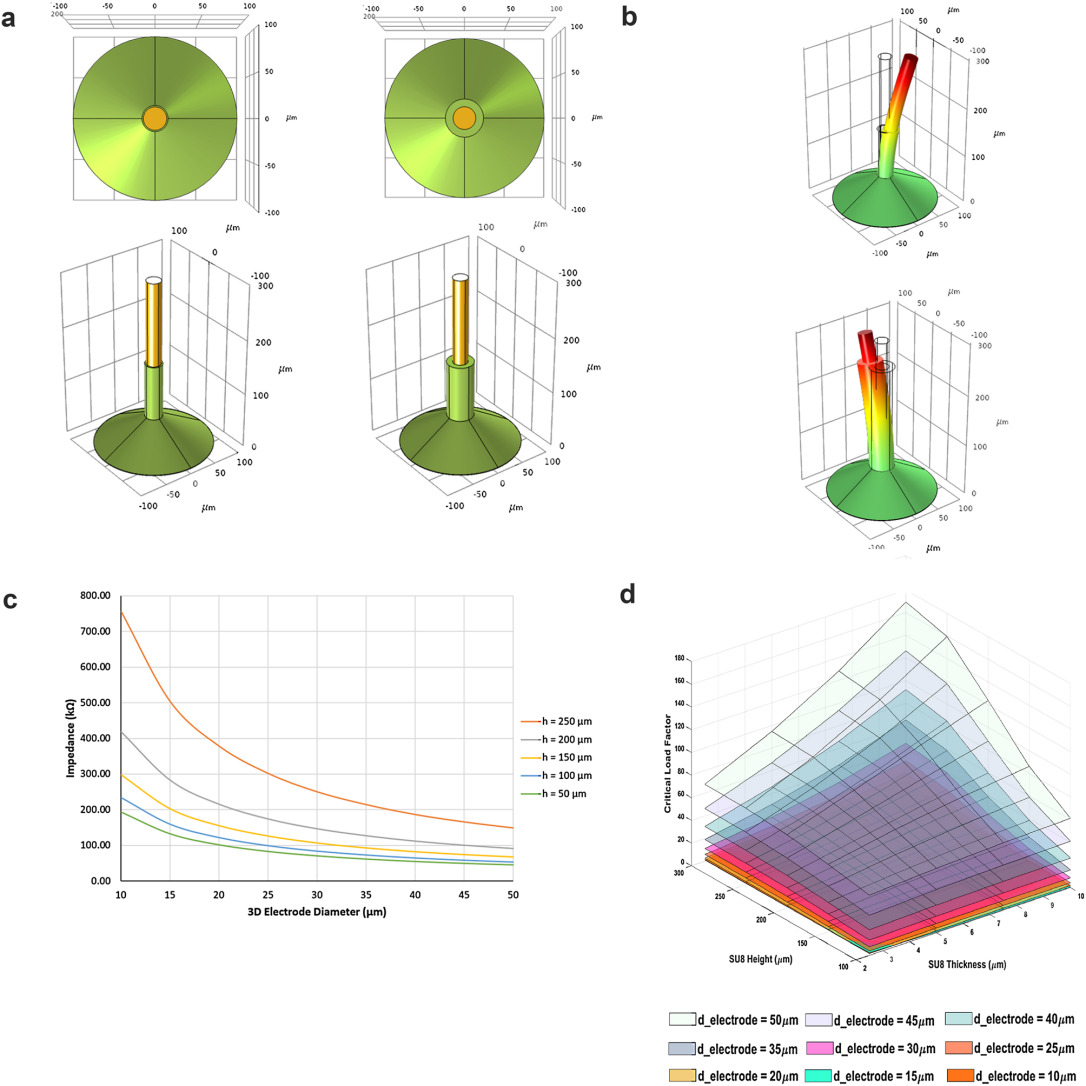
(**a)** Isometric view of two 3D electrodes showing the simulated 3D electrode geometry. The geometry of the green SU-8 insulation layer surrounding the gold 3D electrodes was approximated from the true appearance of a 3D electrode resulting from our fabrication process: a thick base that gradually turns into a uniform coating around the shaft due to the “wicking effect” from surface tension. Both electrodes have the same overall electrode height, SU-8 insulation height, and 3D electrode diameter, but different SU-8 insulation thicknesses. (**b)** Buckling simulation showing two 3D electrodes with different SU-8 thicknesses that are both subject to an identical, arbitrarily chosen, load at the tip. The body of the electrode with thicker SU-8 is deflected less. (**c)** Simulated electrode impedance at 1 kHz for different electrode diameters and insulation heights. Each curve represents the impedance of a 300 µm tall 3D electrode with various diameters of SU-8 insulation at a constant height. The trends in this plot indicate the correlation between uninsulated electrode surface area and the impedance of the electrode. As electrode diameter increases, impedance decreases due to a larger surface area. As the insulation height decreases, impedance decreases due to a larger uninsulated electrode surface area. (**d)** Simulated critical load factors for 300 µm tall 3D electrodes of different diameters, SU-8 thickness, and SU-8 height. Each plane represents a different gold electrode diameter. An increasing gold electrode diameter increases the load bearing capacity of the electrode structure. The load bearing capacity of a particular electrode diameter can be optimized by increasing the SU-8 height and thickness.

Our understanding of brain functions under normal and pathological conditions remains limited, due in large part, to barriers in monitoring subtle electrical signals from large networks of interconnected neurons. As such, the underlying neuronal dynamics of many neurological disorders remain unknown, precluding our ability to treat perturbed brain function using neuro-stimulation approaches^1–3^. This lack of fundamental knowledge stems from our inability to monitor neural activities from complex, synaptically connected networks at a high spatial–temporal resolution, and over extended time periods. In an attempt to fill these gaps, microelectrodes embedded in Multi-Electrode Arrays (MEAs) are now routinely interfaced with a variety of homogeneous cell culture and brain slice preparations maintained in-vitro^4–7^. However, as the neural networks established in culture are artificial, the connectivity patterns studied in any given experiment may vary from preparation to preparation and are likely not representative of in vivo network phenomena. While MEAs are useful in their utility to monitor neural activity simultaneously at multiple sites, this approach is limited in its spatio-temporal resolution^8,9^, even more so when tentatively interfaced with brain slices. The signal-to-noise ratio (SNR) offered by traditional MEAs is often low^10^ and precludes longer-term recording of spontaneously active networks at a resolution, high enough to decipher excitatory and inhibitory synaptic potentials related to neural activity and connectivity. These limitations are due mainly to three factors: (1) traditional planar electrodes used in MEAs only record neural activity from the outer and “traumatized” layers of brain slices, which harbour a larger population of either dead or dying cells^4^; (2) the surface of the tissue damaged during slicing often releases proteases and ions, such as potassium and sodium, which in turn result in excitotoxicity of the adjacent area, thus causing further tissue damage at the recording site resulting in electrical artefacts; (3) the perfusion system required to provide a continuous flow of nutrients and oxygen at a set temperature to maintain the brain slice creates a flow of charged ions within the recording chamber and around the microelectrodes, generating electrical noise^11,12^ (Fig. 1a). Even in those instances where recordings are possible, the above stated challenges make data collection and interpretation inconsistent and unreliable^13^. In contrast, while several types of protruding three-dimensional microelectrodes have been developed to offer alternatives to planar electrodes which record higher fidelity activity in brain slices (e.g. spine-shaped protrusion electrodes^14^, carbon nanotube electrodes^15^ or metal-transfer-micromolded electrodes^16,17^) the efficacy of the recorded potentials is often suboptimal due to tissue damage and low SNR^9^.

Most of the above-mentioned challenges are carried forward in the field of implantable neural electrodes designed for the recording and stimulation purposes. In these situations, the lack of signal fidelity precludes the development of “smart” implants with fully responsive and adaptable feedback loop system. Moreover, current neural interfaces do not provide high enough SNR, granularity or coverage required to generate sufficient and reliable data deemed essential for artificial intelligence and machine learning applications in clinical practices. The existing devices although tend to provide a decent coverage of the cortical brain structures, albeit with low SNR and spatial resolution (e.g. traditional electrocorticograms (ECoGs) used in the evaluation for epilepsy surgery^18^ or higher density alternative MicroECoGs, which suffer from low SNR), or a nominal coverage of the brain with higher SNR and spatial resolution (e.g. Utah Array^19^, Paradromic^20^, Neuralink^21^. Additionally, the implanted electrodes often result in scar formation around the implant and as such not only affect the SNR but also perturb neuronal functions. Ideally, all of the above problems would need to be concurrently solved to maximize our ability to map the brain, develop effective machine learning algorithms that can improve smart feedback loop systems for neuromodulation, and ultimately improve patient outcomes.

Based on an electrical and structural computational simulation paradigm, we describe here a novel fabrication method that enabled the development of a reusable in vitro multisite array of three-dimensional microelectrodes permitting high-resolution, long-term ECoG-like cortical recording with minimal tissue invasion and damage. Unlike most previously published work on microelectrodes, the fabrication process presented here is scalable and easily adaptable for clinical monitoring and therapeutic applications such as intracranial monitoring and brain machine interfaces.

## Results

### Preliminary design and computational validation

A computational simulation was constructed to investigate the spectrum of three-dimensional electrode geometries attainable with our scalable fabrication process. The electrode array performance can be characterized by several interdependent parameters: the neural selectivity, sensitivity, and long-term durability. Whereas the selectivity increases by minimizing the geometric area (GA) of the electrode to achieve more localized recordings, it does nevertheless augment electrode impedance while reducing signal quality and the attenuated sensitivity. The electrode sensitivity can, however, be increased by maximizing the electrochemical surface area (ESA) through surface modifications like conductive coatings, which reduce electrode impedance and improve signal quality^9^. Electrode durability mainly depends on the structure, material biocompatibility and usage such as electrical stimulation, which has proven to reduce sensitivity thereby affecting the longevity of many neurostimulation medical devices^22,23^. Assuming that the material biocompatibility and stimulation protocols have been properly selected to maximize the longevity of the electrodes, optimizing GA and ESA for a given electrode structure and composition is a fundamental requirement of MEA and neuro-implant designs.

In the work presented here, the GA of three-dimensional microelectrodes was controlled by the electrode height and diameter while the ESA was calibrated by the uninsulated electrode surface area. In addition to its insulating properties, an SU-8 (biocompatible polymer) coating surrounding the gold wires was also added to improve electrode strength and durability. To characterize the electrical and mechanical contributions of the SU-8 coating, our group developed two computational simulations using the *Electric Currents* and *Solid Mechanics* modules in COMSOL Multiphysics (COMSOL Inc., Burlington MA). The goals of these simulations were to characterize the electrode impedance and relative mechanical behavior of 3D electrodes in response to axial loading.

The impedances calculated using our model ranged from 45 kΩ to 12 MΩ (Fig. 1c., 50 µm gold wire with maximum ESA and 10 µm gold wire with minimum ESA). We however found relatively small variations in electrode impedance with varying SU-8 thickness. The trend in our simulated impedances indicates decreasing impedance with increasing ESA, which is consistent with trends reported in literature^24^.Furthermore, our simulated impedances are consistent with values reported for commercially available 3D electrodes in 0.9% saline, which validated the accuracy of our simulation^3,4,25^.

This computational analysis also demonstrated that the SU-8 thickness played a prominent role in our mechanical simulations (Fig. 1b). Simulated critical load factors varied between 1.38 and 169, which suggests that thicker SU-8 coatings delay the onset of buckling. The critical load factor is a safety factor describing the ratio between the buckling load to the applied load. The relative trend in critical load factors for the same load applied to different geometries can be used as a proxy for characterizing electrode strength and stability. The mechanical simulations suggested that the SU-8 coating improved the structural strength and stability of our 3D electrodes, which was important for mitigating dynamic factors in an in vitro and in vivo environment and achieving stable recordings.

The results of these simulations provided a basis for future experimental validation of the range of three-dimensional electrode geometries achievable with our fabrication process. Optimizing the SU-8 geometry for gold electrode diameters of 25 µm (~ 1mil) and 50 µm (~ 2mil) was of particular interest as these are considered standard wire sizes in the electronics industry for wire bonding, a common and inexpensive method of creating integrated circuitry connections, allowing for increased scalability. Furthermore, our simulations provided a foundation for optimizing the electrode design in terms of low impedance and mechanical robustness for any custom wire diameter, and/or wire material, thus allowing us to maximize reusability and electrical performance and create durable, high density, and high-performance three-dimensional in vitro electrode arrays.

### Flexible fabrication process of three-dimensional gold microelectrodes for in vitro recordings

**Figure 2:**
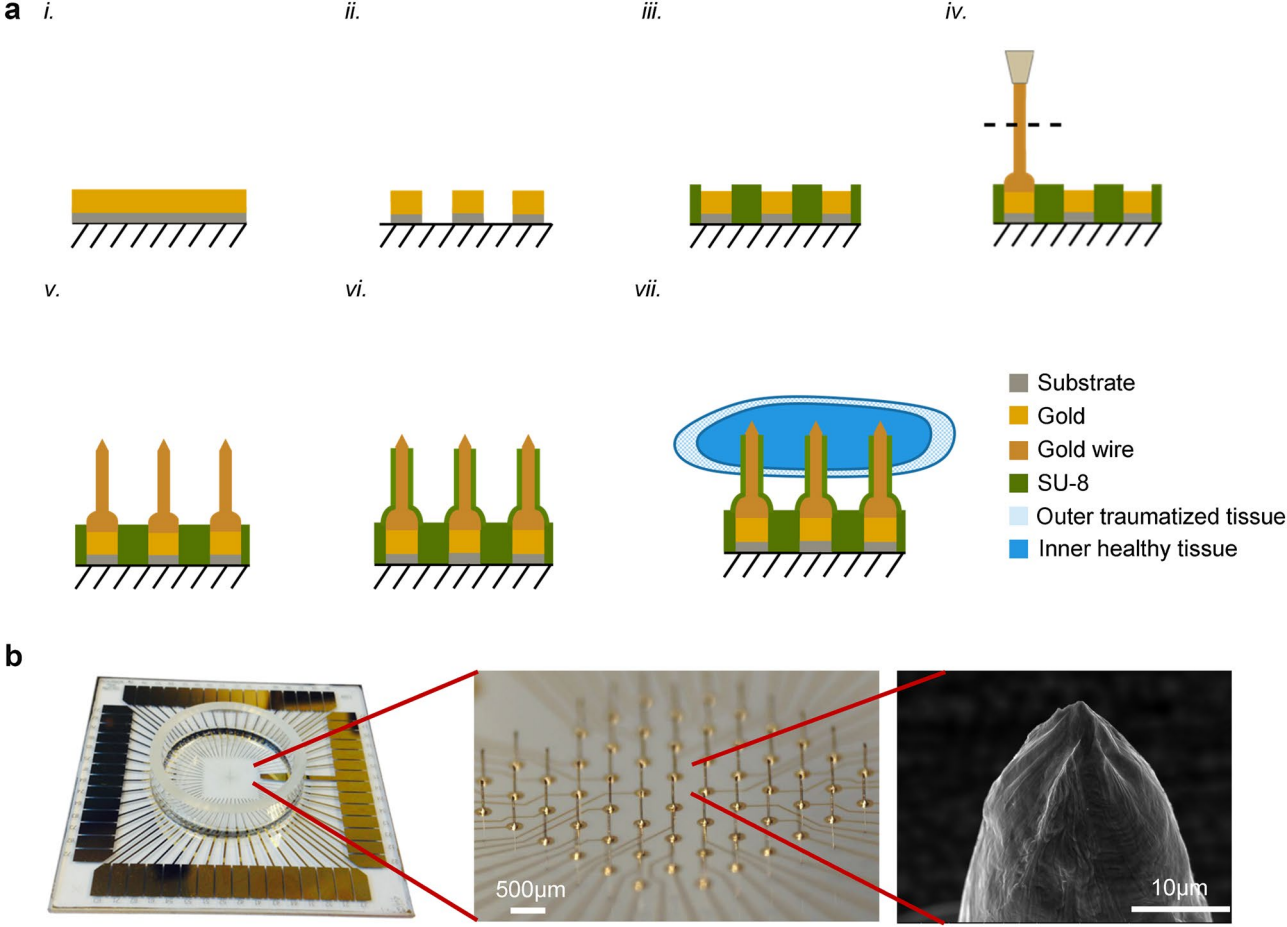
(**a)** Side view schematic of the fabrication process of the three dimensional gold microelectrodes: i to iii, metal electrode deposition and patterning; iv to v, Three-dimensional spike electrode fabrication onto planar electrodes, with the dashed line indicating break height; vi, insulation layer deposition and vii, schematic showing only the bare tips of the electrodes directly interfacing with neural cells located inside the brain slice, i.e. within the remaining healthy tissue. (**b)** From left to right; Left: picture of the whole 49 mm × 49 mm square Multi-Electrode Array chip. The rectangular gold pads on the four sides allow the connection between the recording setup and the three-dimensional microelectrodes present in the center. Middle: magnified image showing a section of the array of gold three-dimensional electrodes under an optical microscope. The three-dimensional microelectrodes shown are separated by a 500 µm gap from each other and are connected to the outer rectangular pads described previously by mean of their respective micro-wires. Right: picture of an uncoated tip from a three-dimensional electrode obtained with a Scanning Electron Microscope (SEM). The electrode diameter and the sharpness of the tip allows for optimal penetration within the brain slice, which limits the invasiveness.

Based on the initial computation and simulation parameters, micro-electrodes were designed and fabricated to record from brain tissue in vitro. Standard gold planar microelectrodes were first fabricated using conventional photolithography techniques^26^ (Fig. 2a, i to iii). Microelectrode sizes and intervals were adjusted according to experimental needs by modifying the photomask designs thus allowing us to keep the design relatively simple, economical, and scalable. Three-dimensional gold microwires were then individually bonded onto the surface of the planar electrodes and coated with SU-8 (Fig. 2a, iv to vi). Only the bases and edges of the microwires were insulated with SU-8, leaving the conductive tips electrically exposed. This method yielded full control and flexibility in the size of each single three-dimensional electrode (diameter and height of wires used), inter-electrode spacing, and materials used (gold in the present example, but palladium, platinum etc. could also be implemented to improve electrical stimulation properties). The electrodes were individually fabricated and electronically addressable. The three-dimensional gold electrodes reported here were purposefully fabricated with a targeted height of 300 μm (average = 266 μm, standard deviation = 27 μm, n = 30). Gold wire diameters of either 17 μm or 25 µm were used. These heights, spacing and materials were chosen specifically for recording from 400 µm thick acute brain slices of mice (Fig. 2a, vii), adjustable to suit any other experimental requirement. Following the fabrication, we characterized and validated the morphological attributes of the spike microelectrodes with optical microscopy and SEM (Fig. 2b).

**Figure 3:**
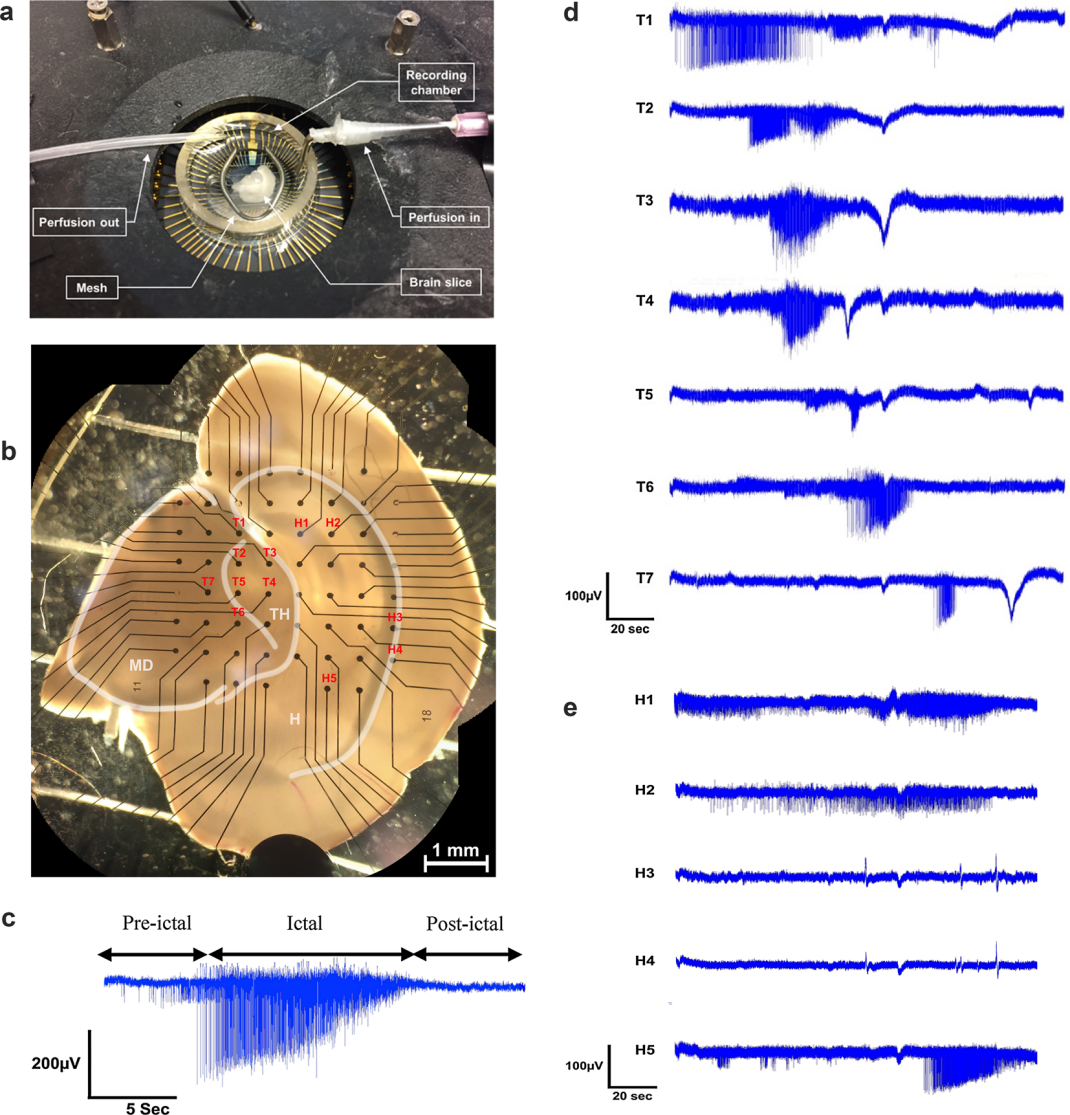
(**a)** MEA positioned into the recording system (MEA1060; Multichannel Systems, Reutlingen, Germany) with an acute hippocampal brain slice positioned at the center of the circular chamber. A mesh is placed on top of the slice to prevent any movement and facilitate the penetration of the three-dimensional electrodes into the slice. aCSF saturated with carbogen at a temperature of 33.0 °C was perfused inside the chamber (right tube) and siphoned at the bath level interface. (**b)** Picture taken with an optical microscope of an acute hippocampal brain slice positioned on an array of electrodes. Areas such as the mid-brain (MD), thalamus (TH) and hippocampus (H) are clearly visible. (**c)** Example of bursting neural field potential recorded with the three-dimensional gold microelectrodes (high 8.5 mM K^+^). Various patterns of activity were recorded, including ictal like events typically seen during seizure-like events. (**d)** Activity recorded simultaneously within the thalamus and mid-brain from the same brain slice. Notice moving ictal timeframes indicative of seizure propagation (**b**). (**e)** Simultaneously, activity was recorded within the hippocampus (CA1 (H3/H4/H5), CA2 (H2) and CA3 (H1)).

After cleaning and sterilizing these three-dimensional MEAs, poly-D-lysine was used as a coating material to enhance the interfacing between electrodes and neurons^27^. Acute hippocampal brain slices (400 µm thick) from C3HeB/FeJ mice littermates (P35) were then positioned with the help of an optical microscope in a recording chamber and anchored onto the electrodes arrays by means of a weighted mesh to prevent movement and to facilitate the penetration of the three-dimensional electrodes into the slice (Fig. 3a,b). Penetration depth was not measured, but is assumed to be consistent across experiments. After positioning mouse acute hippocampal brain slices over the electrodes in an activity triggering artificial cerebrospinal fluid (aCSF, 0 mM Mg^2+^ or high 8.5 mM K^+^), spontaneous neural activity was consistently recorded (n = 55, 98% of the time) in vitro at multiple electrode sites and across the different channels. The recorded activity could be tracked within the entire brain slice and the patterns of propagation were identified (Fig. 3c–e; video available in supplementary material). This activity consisted of bursting ictal events, either localized or spread across the slices, with high frequency activity (~ 80 Hz) often observed in mammalian brain systems expressing hypersynchronous activity^28–32^ (Fig. 3c). This provided us with the opportunity to track high frequency bursting activity between different areas of the brain slice and to analyse its overall excitability. For example, while we anticipated recording neural activity within the well-studied region of the hippocampus (Fig. 3e), we also recorded high-frequency bursting activity in the mid-brain and thalamic regions (Fig. 3d). Unlike previously reported data obtained using three-dimensional electrodes^13^, this bursting activity was reliably and consistently traceable within a brain slice, thus permitting the identification of neural network pathways.

The SNR of the gold three-dimensional microelectrodes was compared with earlier reported devices including well characterized^9,26,27^ traditional planar microelectrodes. These new 3D microelectrodes offered a reduced mean noise of 20 μV (compared to 50–70 μV for traditional planar microelectrodes), which we attribute to the insulating coating present at the bases and edges of the 3D structure. The highest recorded field potential activity peak-to-peak with the 3D microelectrodes was 3.2 mV, compared with signals of < 1 mV with traditional planar microelectrodes^9^. Overall, these new 3D microelectrodes offered a much higher SNR (> 300% increase) than previously reported devices using traditional planar microelectrodes. Finally, as the bases and edges of these 3D microelectrodes were insulated, we recorded activity from within the brain slice where most electrically active cells remain viable^4^, thus enabling continuous recordings over a period up to 4 h. As expected, based on the computational simulation, the insulation on the electrode edges provided structural support, which reduced the physical degradation of the three-dimensional microelectrodes over time and made the MEAs reusable for at least 15 times in an in vitro setting. The MEAs showed no signs of damage after 15 tests, but no further destructive testing was performed. An impedance check is completed by MC_Rack prior to each experiment wherein each electrode is verified to be functional to ensure proper connection and recording capabilities. This serves as a baseline metric for reusability.

We next sorted to determine whether our newly fabricated electrodes would also have utility as implantable devices in-vivo. We therefore transferred the fabrication process to develop implantable flexible substrate for recording induced seizures in freely behaving animals to seek further the electrodes clinical potential.

### Transfer onto flexible substrate for in vivo epilepsy recording

The use of three-dimensional electrode array in-vivo in humans and other animals has been demonstrated in multiple ways, often using devices such as the Utah Array or Michigan Array^19,33^. These penetrating recording tools have been used to improve both the signal quality and spatial resolution simultaneously. Several publications have however reported that 3D electrodes developed on a rigid substrate result in scar formation around the recording or stimulating sites^34,35^. These scars in turn dramatically reduce the signal resolution offered by the recording electrodes and thus affect the longevity of the implantable neuromodulation devices. In addition to recording from only a very limited area of brain slice and therefore not providing enough coverage (e.g. intracranial monitoring for the evaluation of epilepsy surgery), the three-dimensional electrode arrays use has remained limited to research applications and not extended to clinical practice. We deemed it essential that in order to limit tissue scar formation, which is a deterrent to long-term recordings, the substrate would need to be modified and made flexible such as traditionally used ECoGs^36^ in clinical practice, or flexible arrays for animal research^18^. We therefore next took on the challenge of embedding three-dimensional electrode array onto a flexible substrate to merge both properties: high signal resolution and improved longevity.

**Figure 4:**
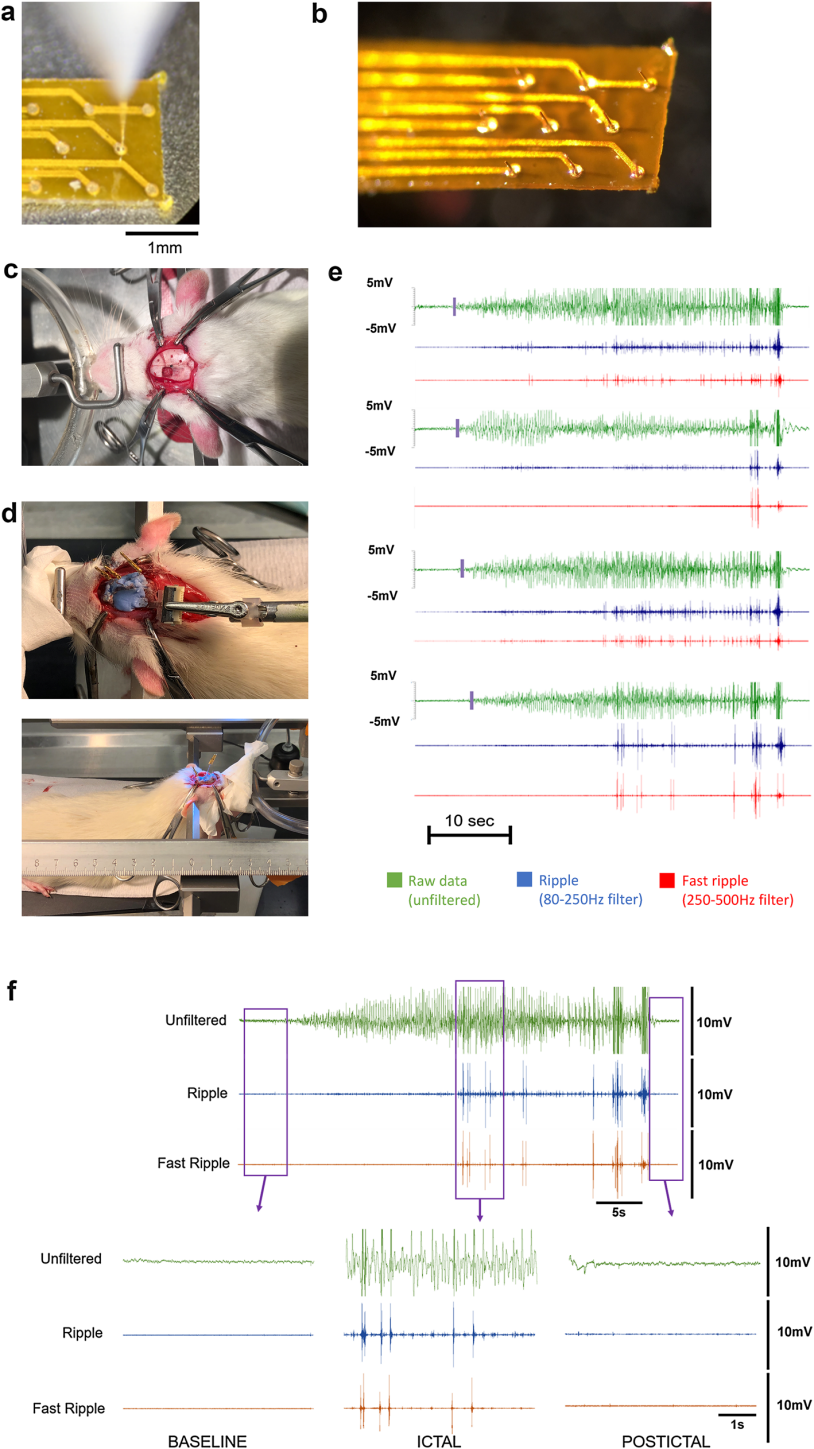
Fabrication process, implantation, and results of in-vivo electrodes. (**a**) Bonding on a planar array of micro-electrodes positioned 350 µm apart to create three-dimensional electrodes. (**b**) Array of three-dimensional micro-electrodes with coating at the base and edges of the structures. (**c**) Adult Sprague–Dawley rats where anesthetized and (**d**) flexible implants were positioned on the motor cortex following a craniotomy. (**e**) Bipolar stimulation electrodes implanted in the corpus callosum of the rat right hemisphere were used to induce seizures through electrical stimulation (electrical kindling) and recorded through Neuraura’s micro-sensors. Only the four channels recording relevant activity are shown here. Raw LFP recordings were bandpass filtered in the HFO frequency ranges using a finite impulse response (FIR) filter. Raw recording are depicted in green, filtered ripple (80–250 Hz filter) in blue, and filtered fast ripple (250–500 Hz filter) in red. Purple markers show the beginning of seizure recording across channels. Amplifier used for in-vivo recordings outputted data in volts. (**f**) Detailed view of one channel showing action potentials during the baseline, ictal and postictal periods.

Adapting a similar fabrication process to the one described in the previous section, we modified the wire bonding parameters to create three-dimensional electrodes on flexible substrates from the printed circuit board (PCB) industry. Following the design of a neocortical implant tailored to record activity from a freely behaving rodent, two surface finishes were tested: electroless nickel immersion gold (ENIG) and electroless nickel electroless palladium immersion gold (ENEPIG). Such processes often use polyimide or polyethylene terephthalate (PET) as a base which has already been demonstrated to be biocompatible and relatively inert^37^. Three-dimensional micro-electrodes with similar dimensions to the one developed on the glass substrate were then added to the flexible substrates (Fig. 4a). No difference in bonding success was noticed between ENIG and ENEPIG. The bases and edges of the electrodes were then coated with SU-8 using a similar process to the one used for the MEA fabrication (Fig. 4b). The animal implants were then rinsed with autoclaved deionized water and sterilized with 70% ethanol.

Adult Sprague–Dawley (SD) rats (n = 4) were anesthetized to proceed with the implantation (Fig. 4c). Holes were drilled into the skull for a ground screw, 2 anchor screws, and a twisted bipolar electrode for chronic implantation in the corpus callosum of the right hemisphere. The bipolar electrode was used to electrically stimulate the rat brain in order to elicit electrographic seizures, a well-established technique called electrical kindling^38^. A small craniotomy was performed to expose the left caudal forelimb area of the motor cortex and the dura was removed to expose the neocortex (Fig. 4c). The flexible substrate with embedded three-dimensional electrodes was then placed on the exposed motor cortex and the entire assembly on the skull was cemented in place with dental cement (Fig. 4d).

Following a minimum 7 days recovery period, an electrographic seizure (after discharge) was elicited with the stimulating electrode using a Grass S88 stimulator (Natus Neurology, Warwick, RI) and the seizure duration measured and severity scored based on Racine’s scale^39^. All rats consistently had a minimum behavioral stage 4 seizure, indicated by a generalized (evolved) seizure involving bilateral forelimb clonus. Simultaneously, local field potentials were recorded through the flexible implant using a custom-made wireless recording unit (Neuraura Biotech Inc., Canada). While the implant could record from up to 8 channels, electrical activity was recorded through four channels at a time in this application at a sample frequency of 1 kHz. Electrical after discharges characteristic of seizure activity were recorded through the three-dimensional micro-electrodes (Fig. 4e,f). The signal-to-noise ratio was again calculated, by taking the ratio of the maximum peak-to-peak amplitude of the signal during seizure onset to the mean noise during inter-ictal periods, (max SNR above 10) and compared to current standards^9,26^, providing confirmation that the three-dimensional electrodes used in-vivo have a SNR significantly higher than traditional stainless-steel electrode traditionally used in research settings. Seizure propagation could also be traced between the three-dimensional electrodes demonstrating that electrographic seizures can be detected at a sub-millimeter level. Resulting from this increased SNR, HFOs were also detected in 100% of recorded seizures with ripples (blue, 80–250 Hz) and fast ripples (red, 250–500 Hz) bands being clearly distinguishable from background activity.

## Discussion

The field of neuroscience and its clinical applications are in dire need to design and develop neural-interfaces and neuro-prosthetic devices that would enable high resolution recordings of brain activity over an extended time period. In this study, we first generated a computational simulation paradigm. This enabled us to demonstrate that the addition of SU-8 with different thicknesses and height around a conductive wire would dramatically affect the electrical characteristic as well as the structural stability of the micro-electrode. As a result, an appropriate balance needed to be realized when fabricating micro-electrodes to optimize the impedance and structural robustness. The ideal electrode would have the lowest impedance while being the most mechanically robust. Based on the results of our simulation (Fig. 1c,d), a 300 µm tall and 50 µm diameter gold electrode with 10 µm SU-8 thickness provides the largest critical load factor and is therefore the most mechanically robust. The impedance of the electrode is a function of the SU-8 insulation height which determines the electrochemical sensing area of the electrode. For a 300 µm tall and 50 µm diameter electrode with 10 µm SU-8 thickness, the impedance values vary between ~ 50 and 150 kΩ for SU-8 insulation heights between 50 and 250 µm. Furthermore, increasing SU-8 insulation heights also resulted in increased critical load factors. Due to the highly compliant nature of our three-dimensional gold micro-electrodes, a larger critical load factor was preferred to make the electrode more mechanically robust. However, as we did not have access to 50 µm diameter gold wire, 25 µm and 17 µm gold wire were used in our experimental work. Because of the highly adaptable and scalable characteristics of this fabrication process, unique parameters could be adapted to meet different experimental and clinical needs. As demonstrated in the in-vivo and in-vitro experiments, the three-dimensional micro-electrodes presented here offer an improved recording capability when considering signal-to-noise ratio, detection of HFOs and traceability of neural activity.

The three-dimensional microelectrodes presented here were fabricated using thermosonic gold wire bonding—a widely used process in the electronics industry. This approach resulted in improved manufacturability and fabrication in comparison to previously reported methods of fabricating 3D electrode arrays like the Utah array. Previously reported methods involved multi-step micromachining processes comprising of multiple stages of material deposition followed by selective material removal^40^. These methods are relatively costly, and inherently susceptible to inconsistencies in the supply chain due to multiple processes involved and the individual complexities of each fabrication step. This hinders process scalability for large scale manufacturing. In contrast, while the wire bonder used in this work was manual, our primarily additive wire bonding process could be performed with an industrial scale fully automated wire bonder which can form between 5 and 12 wires (3D electrodes) per second once fully optimized for a particular electrode geometry, with each electrode as fine as 40 µm apart. With our additive wire bonding process, we could match and/or exceed the electrode density (electrodes per area) of any commercially available in-vitro 3D microelectrode array presently available (to the best of our knowledge^9^). The electrode pitch on the flexible probes used in our in-vivo experiments was finer than previously reported in-vivo flexible 3D arrays, thus allowing higher spatial resolution^41^. The SNR achieved in our in vivo experiments was also much higher than reported for other flexible 3D arrays implanted in the rat motor cortex^41^.

The three-dimensional microelectrodes presented here were fabricated using readily available materials and processes used in the electronics industry and optimized for recording neural signals. Our implanted electrodes not only remained in place and monitored induced seizures but they also did not cause scarring. Although we did not attempt to record spontaneous activity of either individual or populations of neurons but we are confident that these electrodes should be able to pick field potentials from cortical neurons. This may however, require further refinement and the use of gold wires with larger diameter to create gold electrodes; parenthetically, this may also further decrease the impedance. Furthermore, while the electrodes presented here are made of gold for its lower impedance and higher recording capabilities, the fabrication process can also be modified to include other materials that are better suited for electrical stimulation capabilities. For instance, platinum and iridium can be investigated first as these materials are wire bondable, biocompatible and known to be superior to gold for neurostimulation^42,43^. This would however require a wire bonder used in large scale industrial processes, and such was not available for this work. Such an approach will eliminate the need for having to stimulate neurons using external electrodes and could thus also be used to perturb neurons during various behaviors in freely moving animals. Finally, conductive coatings such as poly(3,4-ethylenedioxythiophene) polystyrene sulfonate (PEDOT:PSS) and carbon nanotubes can be applied to three-dimensional gold electrodes to improve the ESA even further while simultaneously improving the charge injection capacity of the electrodes thus optimizing the geometry for stimulation^26,44^ even further.

Finally, because the advances in the field of neuro-technology are currently hampered by our inability to record brain activity at a high SNR and to stimulate neurons safely, many opportunities in the area of neuromodulation and brain machine interface (BMI) can be unlocked due to our improved and flexible neural electrodes. By improving the SNR of the recording electrodes in feedback loop systems, this would facilitate the data cleaning and analysis steps, and therefore permit improved stimulation output of smart neuro-implants. For example, when considering the practice of epilepsy surgery and neuro -stimulation treatment, the electrodes presented in this study can provide 20 × increase spatial resolution versus existing ECoGs, maintain large coverage of brain areas versus the Utah Array, and provide at least 3 × improved SNR compared to traditional ECoGs. In the case of epilepsy surgery, these improved capabilities will enable neurosurgeons to resect pathological tissue from the patients with more precision. This will also facilitate brain mapping and fasten diagnostic process/foci identification, allowing for the full spectrum of neural activity to be studied, including the differentiation between “normal” and “pathological” HFOs^45^. In turn, pathological HFOs have been recognized as potential biomarkers for epileptogenic zones and resection or stimulation of brain tissue eliciting such activity has been shown to lead to better post-surgical outcomes^46,47^.

This new technology allows for a far greater capture of near real time neural activity. With improved recording capabilities, responsive neurostimulation can be further improved, leading to significant impacts in medical implants ranging from epilepsy and seizure management^48,49^, occipital recording/stimulation for visual prosthesis^50^ (Second Sight, US), language prosthetics^51^, or limbs prosthetics^52^. In turn, an improved feedback loop system that stimulates the brain only when required has the opportunity to reduce the formation of scar tissue and improve longevity of implants, making them more widespread and successful (if not more) in a manner analogous to that of a cardiac pacemaker.

## Manufacturing and methods

### Simulation

To reduce computational time, the simulated 3D electrode geometries were treated as cylinders. A 300 µm tall gold cylinder of fixed height and diameters between 10 and 50 µm was used to model the 3D electrode, and a 200 µm diameter gold pad was used to model the electrode base. The SU-8 coating was modelled in two parts to approximate the SU-8 structure achieved with our fabrication process. The approximation consists of a conical lower portion with a height of 50 µm, a fixed 100 µm base diameter and varying upper diameters such that the thickness of SU-8 around the gold wire varied between 2.5 and 10 µm. The upper portion of the insulation was modelled as annular cylinder, continuous with the lower portion, with thicknesses varying between 2.5 and 10 µm, and heights varying between 0 and 250 µm (only a conical SU-8 base present and only the tip of the gold wire is exposed, leaving and area of 20–30 µm).

For the impedance simulation, the 3D electrode was separated 500 µm from a 500 µm diameter × 500 µm tall AgCl counter-electrode and surrounded by a 1 mm diameter × 1 mm tall cylinder of physiological saline (0.9% NaCl). The upper and lower boundaries of the cylinder were assigned insulating boundary conditions (no normal current flow) while the circumferential boundaries were set as ground (V = 0 V) to represent a semi-infinite medium. The counter-electrode was assigned a fixed potential of V = 1 V and a current of 10 nA was applied at the 3D electrode. The properties of the electrode–electrolyte interfacial layer were approximated using literature values for Au in 0.9% NaCl^1^, and electrode impedances were calculated at 1 kHz as the ratio between the RMS voltage and current. For the mechanical simulations, the lower boundaries of the gold wire and SU-8 base were assigned fixed conditions, while the upper boundary of the wire was treated as a free end. The interior Au/SU-8 boundaries were conditioned to prevent penetration between the boundaries and move together during deformation. To characterize the relative trend in load bearing capacity of different 3D electrode structures, an arbitrarily chosen 1 $\frac{mN}{{m^{2} }}$ downwards longitudinal load was applied to the tip of the electrode and the resulting critical load factors were compared. A default physics-controlled fine mesh was used for all cases, with relatively small variations with finer mesh elements.

### MEA fabrication

Planar microelectrodes in an 8 × 8 array configuration were fabricated using standard photolithography onto a 49 × 49 mm, 1 mm thick glass substrate. The glass was coated with ~ 400 nm of gold, deposited on a 50 nm chrome adhesion layer using a sputter deposition process (CMS-18, Kurt K Lesker Co., Pennsylvania, USA). A positive photoresist, (HPR504, Fujifilm, USA) was then spin coated onto the chrome/gold coated glass slide (3500 rpm, WS-650-23B, Laurell Technologies Corp., North Wales, Pennsylvania, USA). The photo resist was soft baked on a hot plate for 90 s (110 °C) and then exposed using a positive photomask (Photoscience Inc., California, USA) using a Mask Aligner (MA/BA 6, Suss Microtec, Corona, California, USA), and developed using Microposit developer 354 (Dow Chemical Corp., Midland, Michigan, US).

A Cr/Au wet chemical etch was performed to define the multielectrode array and any remaining photoresist was removed using acetone. Sizes and intervals between the circular microelectrodes were adjusted according to experimental needs using different photomask designs in the photolithography process. For the work reported here, electrodes of 100 µm diameter, with inter-electrode spacing of 500 µm, were fabricated (Fig. 2a, ii). An epoxy photoresist (SU-8) was then spin coated over the entire electrode array. The SU-8 layer was patterned with a second photomask to leave the planar microelectrodes bare of SU-8 insulator, but with their connecting wires insulated (Fig. 2a, iii).

### Forming three-dimensional microelectrodes

Spike-shaped three-dimensional gold microelectrodes were added onto the surface of the planar microelectrodes using a manually programmable wire bonder (West-Bond model 454647E, West-Bond Inc., USA). These spikes were created by bonding gold wires onto the planar electrodes, manually extending the wires to a set height and then severing them. Finally, a second insulating layer (SU-8) was locally deposited around the bases and on the sides of the newly formed three-dimensional gold microelectrodes, covering approximately 85% of the electrode height, using a micropipette and a manually operated micromanipulator. This process allowed the three-dimensional electrode tips to be left bare of insulator for the last few micrometers, so that they would be in direct contact with the healthy neural cells inside the brain slice. As with the rest of this highly customizable process (substrate footprint, electrode layout, electrode pitch, electrode height, electrode surface area), the amount of surface area in direct contact with the cells could be adjusted depending on experimental needs.

Three-dimensional electrodes were wire bonded to gold planar pads using a WestBond model 454647E manual wire bonder set to ball bonding mode (West-Bond Inc., USA). A 0.7mil (17.78 µm) diameter or 1mil (25.4 µm) diameter gold wire was used. Thermosonic gold ball bonding was chosen, over other biocompatible wirebondable metals like platinum, due to limitations on the model of wire bonder that was used. Ultrasonic power of 0.6 W was applied for a period of 200 ms. After bonding gold wire to a planar gold pad, the bonding capillary was moved upwards 350 µm and the wire was severed manually using microscissors. This process was repeated to create each three-dimensional microelectrode.

### MEA washing, cleaning and sterilization procedure

After inspection for any defects or debris under an optical microscope, MEAs were rinsed extensively (30–50 times) with filtered, autoclaved distilled water. This ensured complete removal of any fabrication residues deemed harmful for biological tests. Also, following every experiment, MEAs were washed in a similar way to remove cell residues and previous coatings. To completely remove any cell residues, MEAs followed a cleaning procedure using a 10% tergazyme solution. Again, MEAs were extensively rinsed with filtered, autoclaved distilled water to ensure complete removal of any enzymatic solution that would be considered harmful to the cells. On rare occasions, diluted household bleach (5.25% Sodium Hypochlorite diluted with a ratio of 1:500) was used to remove residues or potential fungus. MEAs were then sterilized with 70% ethanol for 30 min and rinsed 3 times with filtered autoclaved distilled water.

### Mice and brain slices

C3HeB/FeJ mice were purchased from Jackson Laboratories (Bar Harbor, ME, U.S.A.), and the colony was maintained in the Animal Resource Facility at the Cumming School of Medicine, University of Calgary. Mice were given food and water ad libitum and kept on a 12-h light/dark cycle. Wild type (+ / +) mice at P35 (postnatal day 35) were used in this study and all procedures in this study were performed in accordance with the recommendations in the Canadian Council for Animal Care. All animal research protocol of this study were approved by the Health Sciences Animal Care Committee of the University of Calgary.

A detailed protocol is published elsewhere^53^. Briefly, on the day of the experiment, animals were anesthetized (Ketamine–Dormitor mixture; 0.1 ml/100 g; i.p.), sacrificed, and their brains were removed and quickly placed into ice cold, oxygenated (95% O2 / 5% CO2) artificial cerebrospinal fluid (aCSF; all in mM: 86 NaCl, 3 KCl, 4 MgCl2, 1 NaH2PO4, 75 sucrose, 25 glucose, 1 CaCl2, and 25 NaHCO3). Coronal slices (Approx. 400 μm; 1.5 to − 0.3 mm relative to Bregma) containing the hippocampus were prepared using a Leica VT 1000S vibratome. These slices were then placed in aCSF containing (in mM) 124 NaCl, 4.5 KCl, 1 MgCl2, 10 glucose, 1 CaCl2, and 26 NaHCO3 at 32 °C for 30 min to recover, and remained afterwards in a aCSF bath at room temperature (22–24 °C) until used. All solutions used during this process were maintained at pH 7.4 and bubbled with 5% CO2 / 95% O2 (carbogen).

### Neural activity recordings with MEAs and analysis

Neural activity was recorded by an MEA amplifier and PCI acquisition card (MEA1060; Multichannel Systems, Reutlingen, Germany) and visualization was made using the software MC_Rack and MEA_Select (Multichannel Systems, Reutlingen, Germany). Software TCX-Control was used to control the temperature regulator and heated cannula (respectively TC02 and PH01, Multichannel Systems, Reutlingen, Germany). The recordings were compiled and processed using MC_DataTool and MC_Rack software respectively. More precisely, a spike detector present in MC_Rack allowed the extraction of timestamp associated with each individual action potential. These outputs were then imported and processed by Excel (Microsoft; Redmond, WA, USA) to better analyze activity frequency or the time elapsed between two adjacent action potentials, known as inter-spike intervals (ISI). Noise was measured using the baseline recording in an aCSF solution without the presence of a brain slice where the electrodes were left for approximately 5 min. The maximum peak-to-peak amplitudes were then analyzed to calculate the signal to noise ratio.

### Implant fabrication and preparation

Implants were first designed using Altium Designer (Altium Ltd., NSW, Australia), a common software in the printed circuit boards (PCB) industry. The substrate footprint was approximately 1.75 mm × 8 mm and designed to fit within the available surface area on the motor cortex of Adult Sprague–Dawley (SD) rats. Electrode pads were designed to be 150 µm diameter and interelectrode spacing was defined as 350 µm to accommodate our 8-channel data acquisition hardware. The design can be adapted to increase the number of electrodes and density depending on the number of channel inputs offered by the amplification system being used. The design was sent to a flex PCB manufacturer for fabrication. Using polyimide as a substrate, two processes were followed: electroless nickel immersion gold (ENIG) and electroless nickel electroless palladium immersion gold (ENEPIG), both of which are surface finishes intended to provide a gold wire bondable surface for 3D electrode formation to be added on top of the planar electrode pads. ENIG applies a layer of nickel over the copper, followed by gold over the nickel. ENEPIG applies a layer of nickel over the copper, followed by a layer of palladium over the nickel, followed by a layer of gold over the palladium. Once received by our facility, the fabrication of spike-shaped three-dimensional gold microelectrodes and coating of their edges and bases was done following the same protocol as for MEA fabrication.

### Rats and surgery

Adult Sprague–Dawley (SD) rats (n = 4) weighing 252–300 g at the time of surgery were used in this study. All rats were obtained from Charles River (Quebec, Canada) and experiments were approved by the Health Sciences Animal Care Committee at the University of Calgary. Experiments were carried out in compliance with the ARRIVE guidelines. Rats were maintained on a 12-h light/dark cycle with lights on at 7am. All experiments were conducted during the light phase. Rats received food and water ad libitum.

Rats were initially anesthetized with isoflurane at 5% and maintained at 1.5–2%^54^. The level of anesthesia was monitored by assessing breathing rate and a withdrawal reflex from a light foot pinch. Lidocaine 2% (20 mg/kg, volume of 0.5 ml) was injected subcutaneously at the incision site. A single injection of buprenorphine (dosage of 0.03 mg/kg, volume of 0.05 ml subcutaneous) and baytril (10 mg/kg, 0.2 ml subcutaneous) was given prior to surgery. In addition, twice per day for 3 days post-surgery, animals were individually given buprenorphine jello (oral dose of 0.17 ml); and once a day for 3 days post-surgery, animals were given Baytril. Holes were drilled into the skull for a ground screw (placed above the cerebellum), 2 anchor screws (located in proximity to craniotomy to provide structural stability), and a twisted bipolar electrode (Teflon-coated, stainless steel wire, A-M Systems, Sequim, WA) crimped with gold male amphenol pins with a tip separation of ~ 1 mm for chronic implantation in the corpus callosum of the right hemisphere (− 5.2 mm anterior, 1.0 mm lateral, − 2.5 mm ventral). A small craniotomy was performed to expose the left caudal forelimb area of the motor cortex (2 mm anterior from bregma, 2 mm posterior, 3 mm lateral from midline). The dura was removed to expose the cortex. Body temperature saline was used to keep the exposed cortex moist. The flexible 8 channel cortical grid electrode measuring 1.75 mm × 2 mm was placed on the exposed motor cortex. The entire assembly on the skull was cemented in place with dental cement. An FFC connector and standard tip jack connectors (Cinch Connectivity Solutions Johnson) were used to connect the array to the recording system. A minimum of 7 days was provided for recovery before experiments began.

### Electrical kindling

An electrographic seizure (after discharge) was elicited with a stimulation (60 Hz biphasic square wave; 1 s train; 1 ms pulse widths) through the electrode in the corpus callosum using a Grass S88 stimulator (Natus Neurology, Warwick, RI). The seizure duration and severity observed were recorded based on Racine’s scale^39^. All animals consistently had a minimum behavioral stage 4 when kindled.

## Supplementary Information

Supplementary Information 1.

Supplementary Video 1.
