# Supplementary material for: Three dimensional microelectrodes enable high signal and spatial resolution for neural seizure recordings in brain slices and freely behaving animals
Source: Sci Rep. 2021 Nov 9;11:21952. doi: 10.1038/s41598-021-01528-4 (PMC8578611; doi:10.1038/s41598-021-01528-4)
Supplement: Supplementary file 1 — Supplementary Information 1. [file 41598_2021_1528_MOESM1_ESM.docx]

Supplementary Materials

**Figure S1**: A detailed view of the in vivo microelectrode array showing dimensions and spacing of the electrodes


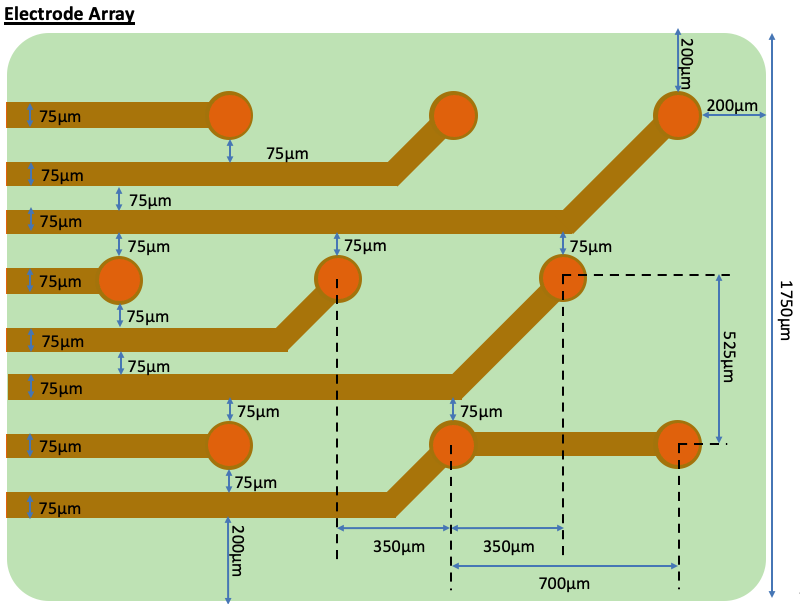


**150µm**

**Table S1**. Comparison of the three-dimensional in vivo microelectrodes developed in this work to other works

|  | **Clinical ECoG* [1]** | **Xiang et al.**  **[2]** | **This Work** |
| --- | --- | --- | --- |
| **Substrate** | Silicone (Silastic™) | Polyimide | Polyimide |
| **Metallization** | Pt, stainless steel | Cr/Au | ENIG, ENEPIG |
| **Electrode Pad** | 3-5mm  (diameter) | 500µm x300µm  (rectangular) | 150µm  (diameter) |
| **Electrode Tip Diameter** | N/A | 80-200µm | 17 or 25µm |
| **Electrode Height** | N/A (planar) | 500-2250µm | 300µm |
| **Electrode Spacing** | 5mm | 0.8mm | 0.35mm (in vivo) |

*Ad-Tech 4-channel strip (Catalog# IS04R-SP10X-000)

[1] V. Palys, “Intracranial Electrodes (icEEG),” *Viktor's notes for the neurosurgery resident*, 13-Jan-2021.

[2] Z. Xiang et al., “A flexible three-dimensional electrode mesh: An enabling technology for wireless brain–computer interface prostheses,” *Microsystems & Nanoengineering*, vol. 2, no. 1, May 2016.

Supplementary Video: A video of a brain slice recording using the gold three-dimensional electrodes can be seen at the following link: link to be provided for journal readers.
